# Buffalo Medical and Surgical Association

**Published:** 1889-09

**Authors:** 


### BUFFALO MEDICAL AND SURGICAL ASSOCIATION.

REGULAR meeting, July 2, 1889,—the President, Dr. ALVIN A. HUBBELL, in the chair.

Dr. HERMAN MYNTER reported and exhibited cases as follows:

CASE I.—*Excision of Elbow-joint.*—Minnie Wett, 18 years of age, entered the Buffalo General Hospital on February 14, 1888. When nine years of age, she injured her left elbow, but recovered perfectly. Since then, however, the arm has several times swelled, and the joint been painful for a time. Two weeks before admission, without known cause, the elbow began to swell, became tender and painful. On examination, a diffuse swelling of the elbow was seen; movements, especially extension, were impaired and painful, some lateral movement possible.

The joint was punctured, irrigated with carbolic acid, and kept in a plaster-of-paris bandage for four weeks. As no improvement followed, arthrectomy was performed on March 24, 1888, by aid of two lateral incisions. The whole capsule was found studded with tubercles, the cartilages more or less ulcerated and absorbed. The epiphyses were in a state of osteo-porosis verging on caries, but were left intact, while the whole capsule was removed.

The wounds were closed with sutures, and an attempt was made to heal them with Schede's blood-clot method, but necrosis occurring in the epiphyses, they were, on May 5th, made to protrude through the incisions, and sawed off.

The cavity was thereafter filled with iodoform-gauze under an antiseptic bandage. Passive motions were used freely and early. She was discharged in July, recovered. She is now in perfect health, and the function of the joint is so perfectly restored that she is able to perform the various duties of an only servant in a large family. Flexion, extension, pronation, and supination are as normal as on the other side.

Of the different methods for excision of the elbow, I prefer two lateral incisions to Langenbeck's long vertical incision. It gives more space than Langenbeck's, while it does not damage the insertion of the triceps and leave scars, which may endanger the movement of the new joint, as in Moneau's and Raux's operations.

CASE II.—*Excision of Hip-joint.*—Patrick Calahan, aged 11, entered the Sisters' Hospital on July 10, 1888. Family history good. For three months he had complained of lameness of right foot, pain in knee and hip. The right leg was flexed in the hip at an angle of 60°, abduced and rotated outwards. Pain on slight movements; no crepitation. Under narcosis, the leg was straightened and put in Volkman's splint, with nine-pound weight, for a few weeks. An abscess formed in Scarpa's triangle, and on August 25th it was opened by incision between the femoral artery and the Sartorius muscle, and three ounces of pus evacuated. Contra-opening was made on inner side of thigh. As no improvement followed, the joint was resected on September 11th by aid of posterior incision, the section being made above trochanter minor. The head, neck, and trochanter were found in a state of caries. He left the hospital in November, 1888. Recovery was retarded by a periostitis, produced by a fall after he had commenced to walk fairly well, but the incisions are now all but healed, the movements in the new joint free and painless, and he walks well with a crutch and a stick, and will, probably, in a short time be able to walk without any support.

CASE III.—*Excision of Ankle-joint.*—Willie McAuliffe, 10 years of age, entered the Sisters' Hospital on August 21, 1888, with tubercular arthritis of right ankle-joint. Poor family history; his mother, one brother, and one sister having died of phthisis.

He received a slight injury on the ankle-joint two years previously; the joint swelled and was very painful; atrophy of calf took place, and he has been unable to walk since. At admission, he could bear but little weight on the foot, and pressure on the plantar surface caused intense pain. There was slight lateral motion present in the tibio-astragaloid articulation with crepitation. Diffuse swelling around the joint, most pronounced behind.

August 24th, arthrectomy of right ankle-joint by transverse anterior incision. The capsule was found studded with tubercles, the cartilages lay loose in the joint, and on posterior surface of the astragalus a small carious cavity was found. The capsule was removed with scissors, the cavity scraped with sharp spoon, the tendons thereafter united with catgut sutures, the wound closed, except in the lateral parts, and the wound healed by Schede's blood-clot method under an antiseptic and a plaster-of-paris bandage. On September 22d the primary bandage was removed, and the wound found healed by first intention. Considerable pressure could be borne without causing pain. He left the hospital the same day. He is now able to walk with ease, and, with the exception of some stiffness in the ankle-joint, considerable atrophy of the calf and the scar, has no traces left of his disease.

This case shows the advantage of anterior incision, by which alone it is possible to remove everything diseased, and of Schede's blood-clot method, by which healing under one bandage is possible. The operation is rare, and has probably not been performed in Buffalo before. I therefore call your attention to it as deserving your consideration in chronic tubercular arthritis of more or less recent occurrence.

CASE IV.—*Total Extirpation of Tongue.*—William Bell, aged 60, was admitted to the Sisters' Hospital on June 4, 1889, with cancer of tongue and floor of the mouth. It was first noticed three months ago, has since been growing rapidly, and has occasioned great pain and emaciation. By inspection a large cancerous ulcer was seen occupying the lateral sides of the whole anterior portion of the tongue and the whole floor, including the sublingual glands. The last posterior inch of the tongue seemed normal.

June 6th, extirpation of the tongue, after Syme's method. After preliminary tracheotomy, the pharynx was plugged with a sponge, attached to a thread, and the lower lip was severed by an incision extending backwards to the hyoid bone. The maxilla inferior was thereafter cut through with a chain saw, and widely separated. With the greatest ease, all the diseased tissues on the floor of the mouth, including the glandulæ sublinguales, could now be separated with scissors from the healthy tissues. The tongue was, thereafter, split in two, and each half pulled out and severed gradually with scissors about one inch in front of the epiglottis, particular care being used in catching the lingual arteries with a hemostatic forceps, before they were cut through. The maxilla inferior was thereafter united with a silver wire, the holes for which were drilled before the bone was cut through; a drainage-tube was led out through the submental region, and the floor of the mouth packed with iodoform-gauze for a couple of days. The patient was fed with the stomach-tube for two weeks. The tracheotomy-tube was removed on June 14th, after which the wound in the trachea rapidly healed. The only after-treatment used was hourly irrigations of permanganate of potash solutions. He has not left the hospital yet, as, so far, there is no union of the maxilla inferior, but he is perfectly able to present himself for your inspection to-night, swallows with comparative ease, has no pain, and has increased considerably in weight.

Extirpation of the whole tongue has always been looked upon as an operation of greatest magnitude, particularly on account of the bleeding; on account of broncho-pneumonia and gangrene of lung from absorption and drawing down of putrid fluids into the lungs; of suffocation from falling back of the tongue, of edema of the glottis, etc. The preliminary tracheotomy relieves us, in a great measure, of these dangers, and the section of the maxilla inferior makes it possible to perform the operation, so to speak, extra ore. The wound, being made with sharp scissors instead of with the écraseur, will be less apt to inflame and be followed by cellulitis and pyemia. If the floor of the mouth is involved, the operation, which I performed (Syme's), is the only one by which all diseased tissues can safely and surely be removed.

CASE V.—*Excision of half of Lower Jaw.*—William Templeman, aged 37, entered the Sisters' Hospital on January 30, 1889, with a fibro-sarcomatous growth of the posterior part of right maxilla inferior. He had formerly had a cancerous growth of the lip removed with a plaster, by a cancer-doctor in Rome, N. Y.

The growth was removed by partial resection of about two inches of right maxilla inferior, and the patient left the hospital recovered in twelve days. He reëntered the hospital on June 3, 1889, with a large relapse, the size of a goose-egg, between the previous sections of the bone and extending forwards and backwards for a considerable distance along the body and ascending ramus of maxilla inferior. Besides, the whole right sub-maxillary gland was cancerously infiltrated. On June 3d, the whole right maxillary bone, with the growth attached, was removed. As a large part of the skin covering the tumor was infiltrated, it was removed too. Thereafter the sub-maxillary gland was extirpated. The large defect was covered by a plastic operation, the flap being brought up from the neck. The patient left, recovered, in eleven days, with the exception of the unavoidable paralysis of the seventh nerve.

CASE VI.—*Traumatic Loss of Biceps.*—John Hubner, aged 27, entered the Sisters' Hospital on February 20, 1889, with a very peculiar lesion, which I do not know has ever been described before. He was caught by a rope and drawn into a cog-wheel, which, besides producing a great many lacerated and torn wounds, completely tore out the biceps muscle of his right arm, severing it in the center and leaving the upper and lower half attached only by their tendons. The tendons were cut over, the dead muscle removed, and the enormous wound healed by skin-grafting. The biceps is the principal flexor and supinator of the forearm, and we should, therefore, expect a considerable or almost complete loss of these movements. I am astonished to see how well he can, nevertheless, flex and supinate his forearm by aid of musculi brachialis internus, pronator teres, and supinator longus as flexors, and supinator brevis as supinator. Remarkably enough, he scarcely seems to notice his loss.

CASE VII.—*Radical Cure of Hernia by McBurney's Method.*—In *New York Medical Record* of March 23, 1889, Dr. McBurney proposes a new operation for the radical cure of inguinal hernia, which, it seems to me, is based upon correct principles, and has much in its favor. The important point in the radical cure of inguinal hernia is the complete removal of the sac and "the restoration to the peritoneal surface of the abdomen, at the point where the hernia begins, of the same smooth tense condition that exists at other parts of the inner lining of the abdominal wall."

Therefore, the peritoneal laxity at the internal ring, which forms the predisposing cause, must be removed and the peritoneum supported at this point.

In order to do this, McBurney splits the canalis inguinalis up to the internal ring, dissects the sac loose to this point, opens it, reduces the contents and then ligates the sac with strong catgut high up on the level of the internal opening, and cuts the rest off. In order to secure support to the peritoneum, he advocates an open treatment of the wound, in order to secure granulations and a dense cicatricial tissue throughout the whole length and depth of the canal.

Simply sewing together the pillars of the external ring, as usually has been done, is an imperfect method, as it still leaves room for an incomplete hernia above the closed external ring, and the hernia may, therefore, again find its way down. Even if silver wires are used, they will cut their way through on account of the continued tension of the divergent fibers of the pillars, and if catgut sutures alone be used they are so soon absorbed that the hernia quickly returns. McBurney's aim, to insure the formation of granulations from the very bottom of the wound to the surface, is not accomplished by simply packing the wound, as the packing easily may become displaced. He, therefore, inserts four to eight stout silk stitches, which bind together the tissues, and keep the wound patulous. The upper row binds together the conjoined tendon, the aponeurosis of the external oblique (including the inner pillar of the ring) and the skin. The lower row of stitches unites the skin and Paupart's ligament, including the lower part of the anterior pillar.

As the wound thus arranged would be unnecessarily wide, he adds, after having packed it down to the very bottom with iodoform-gauze, a couple of heavy tension sutures across the wound, by which also the tension on the edge stitches is relieved, so that they do not cut their way out too soon. Over the whole an antiseptic bandage is applied, and the dressing is changed every four or five days, till the wound is healed, which generally takes about six weeks. The longer time used by this treatment has its compensation in the fact that the patient has not afterwards to use any truss. I have lately performed this operation, and will here show you the patient.

Thom' Saunders, aged 55, has had a rupture, and worn a truss, for many years. It came out on May 25, 1889, and soon became strangulated. Failing in reducing it under narcosis, I, six hours after, made herniotomy, and combined the operation with McBurney's method for radical cure. A large part of the omentum, which was adherent, was cut off and returned. The gut was intensely congested, but else in good condition. The convalescence was complicated by an attack of delirium tremens, during which he ran all over the house, tried to climb the walls, and did everything to prevent a successful result. The wound is now healed, and, as you will see, no impulse, even by coughing, is produced.

Dr. ALVIN A. HUBBELL reported the following

### CASE OF PARALYSIS OF BOTH FACIAL AND BOTH ABDUCENT NERVES, FOLLOWING INJURY.

Henry M.[1]———, aged 23, switchman. On August 21, 1888, while coupling cars loaded with lumber, the young man's head was caught between the ends of the lumber projecting beyond the plat-

1. I am indebted to Dr. C. M. Daniels, Surgeon to the Emergency Hospital, for the privilege of reporting this case, and to Dr. E. H. Norton, Surgeon to the Fitch Accident Hospital, and Dr. C. Kelley, Jr., interne to the Emergency Hospital, for the early history of it. A. A. H.

forms of the two cars between which he stood. He was, afterwards, picked up in an unconscious state, and taken to the Fitch Accident Hospital. From the hospital memoranda we learn that "a scalp wound was inflicted on the left side of the head, over the occiput, and about two inches in length; profuse hemorrhage from the ears, nose, and mouth, with frequent vomiting of blood; pulse, 54; respiration not disturbed." The diagnosis was, "fracture of the base of the skull."

On the following day, August 22d, he was transferred to the Emergency Hospital, and placed under the care of Dr. C. M. Daniels, surgeon to the Erie railroad. He was, at this time, in a semi-conscious state, from which he could be aroused with difficulty. His pulse was about 60, and temperature 90° F. (?) On the left side of the head, just above and behind the ear, there was a semi-circular wound of the scalp, of considerable extent, passing from above, downwards and backwards. The wound was said to be four inches long, although at the Fitch Accident Hospital it was noted as being two inches. The bone beneath did not seem to be fractured. The wound was sutured with catgut, and dressed antiseptically. On the right side, there was slight laceration and much contusion and swelling from above the mastoid process to the clavicle, and the parts were painful and sore.

There was some bleeding from both ears, and as the patient became conscious, it was found the hearing in both was somewhat impaired, most, however, in the right, although ordinary conversation could be heard with ease. There was complete facial paralysis on both sides, every muscle of expression being perfectly immobile, the patient being unable to close the eyelids or move the lips. He could, indeed, "Laugh and cry as from behind a mask," neither smile or frown having any expression in the face. Both right and left abducent nerves (nerves supplying the external recti muscles of the balls) were also completely paralyzed, producing paralytic convergent squint of both eyes. The physiognomy of the patient was thus rendered most peculiar and striking. The vision was a little "hazy," or "smoky," in both eyes, but worse in the right. In the right eye, the accommodation was also impaired and the pupil slightly dilated. The right side of the tongue was dry and rough, and there was total loss of taste, according to the statements of the patient. There was difficulty in swallowing, the muscles of deglutition (probably the palatal muscles) on the right side appearing to be most impaired in function. The patient succeeded better in the act by turning his head to the left side. Only liquids could be swallowed at first. Paralysis of any other part of the body, either of motion or sensation, could not be detected.

The patient, at this time, complained of intense pain in the frontal region of the head, in the eyes, and in the face. This was paroxysmal, coming and going at short intervals.

On the next day, August 23d, there was slight rise of temperature and increased frequency of pulse, and the parts injured, together with the face, were much swollen, and the face was also very red. The pains continued to be severe, requiring the administration of anodynes.

The subsequent history of the case is one of slow improvement. The pains gradually subsided. The patient was able to swallow semi-solid food on the third day, and soon afterwards solid food. The wounds and contusions healed rapidly. The sense of taste had fully returned within a month after the accident. Hearing improved, and the vision, together with the size and reaction of the right pupil, seemed to be normal after a few weeks. A few days after the patient was brought into the hospital, I was asked to see him, and have had occasion to examine him frequently since, and I found the condition of the eyes and ears as above noted. In addition, careful examination of the fundi of the eyes and of the membrana tympani showed no abnormal appearances whatever.

The facial paralysis, at the present time, has so far improved that the patient can nearly close his eyes, and in laughter there is some muscular movement about the mouth and nose. The left abducent nerve has nearly, or quite, regained its function, the left eye moving normally in all directions. The right abducent, however, is still fully paralysed, and the right eye cannot, therefore, be turned to the right. The hearing of the left ear is normal, while that of the right is slightly impaired. Otherwise than the paralysis of the facial and abducent nerves which remains, and the impaired hearing of the right ear, the patient feels that he is as well as before the accident.

Such a combination of symptoms as appears in this case must be exceedingly rare, from whatever cause. It affords an interesting study to determine the character and the locality of lesions that can thus coincidently produce a bilateral paralysis of the facial and abducent nerves, and also more or less transiently affect the sense of taste, the secretions of the mouth, deglutition, the ciliary muscle and iris of one eye, and the senses of vision and hearing.

Fracture of the base of the skull, extending into the petrous portion of each temporal bone, might have been such as to cause paralysis of both facial nerves, but such an injury would, undoubtedly, have also involved other structures so extensively as to terminate the life of the patient. At least, the auditory apparatus would have been so much injured as to produce total deafness. Certainly the abducent nerves could not, almost alone, have been the only structures implicated with the facial. There is no evidence that there was an external injury at or near the point of exit of the facial nerve from the cranium, especially on the left side, sufficient to produce the facial palsy; and, even if there were, the consecutive palsy of the abducent nerves could not thus be accounted for. Neither does it seem probable that a hemorrhage at the base of the brain, within the membranes, could have destroyed the function of both the facial and abducent nerves on both sides, and in their widely different courses, without seriously affecting other nerves and other functions. Nor could there have been a coincident hemorrhage in the pons Varolii or other parts in the course of the paralysed nerves on both sides, without marked paralysis elsewhere.

Where, then, could a lesion take place that would be most likely to destroy the functions of these two pairs of nerves, and at the same time cause the other symptoms? As is well known, the nuclei of several of the cranial nerves are grouped together in the medulla oblongata, beneath the floor of the fourth ventricle of the brain. In the upper part of this region, very near the median line, is the nucleus of the abducens. Just outside of this, and a little below, is the nucleus of the facial. (These nuclei were, not long ago, supposed to be one.) The fibers from the facial nucleus pass inwards and loop around the inner and upper sides of the nucleus of the abducens. Thus there is an intimate relation between the root-fibers of the facial and the nuclei of the facial and abducens.

Therefore, a lesion — for example, a hemorrhage — occurring near the median line in this region could impinge upon or affect the nuclei and fibers of these two nerves on both sides, and very slightly, or not at all, involve other structures. Granting that there was fracture of the base of the skull; granting that there was more or less hemorrhage from the meninges of the brain, yet I believe that the symptoms in this case can be most rationally accounted for on the supposition of a hemorrhage near the median line of the floor of the fourth ventricle, between and affecting the nuclei of the abducent, and the nuclei or root-fibers of the facial nerves. Pressure on nuclei and fibers in near proximity could lead to the less pronounced and less persistent symptoms, such as loss of taste, impairment of vision and hearing, etc., already mentioned.

Dr. M. HARTWIG thought that the lesion in Dr. Hubbell's case ought to be located at the apex of the petrous portion of the temporal bone, probably on both sides.

Dr. FALK believed the lesion to be located in the pons Varolii.

Dr. J. W. PUTNAM agreed with Dr. Hubbell in placing the lesion in the medulla oblongata, near the floor of the fourth ventricle, because of the respiratory symptoms.

Dr. ALVIN A. HUBBELL also reported a case of

### TOTAL MONOCULAR OPHTHALMOPHGIA, EXTERNA AND INTERNA, CAUSED BY INJURY.

Charles Wolf, aged 22, presented himself to me on May 28, 1889, with the following history: About three and one-half weeks ago, while somewhat intoxicated, he had a quarrel with another man, who "punched" him in the left eye with an umbrella-stick. The next day or two afterwards he was admitted to the Toronto Hospital, where he was under the treatment of Dr. G. S. Ryerson. I have received the following note from Dr. Ryerson regarding the case, dated June 6, 1889:

Charles Wolf presented himself at the hospital about three weeks ago with a slight scar in the lower lid, and protrusion of eye; lid showed a little ecchymosis; ophthalmoscopic signs negative; said he was quite blind; went out in about the same state, except that the protrusion had diminished somewhat.

On examining the case I found an irregularly shaped scar on the left lower lid about three-fourths of an inch long, and running from near the inner canthus downwards and outwards. There was no ecchymosis of skin or ball, or other evidence of a very recent injury. There was complete ptosis of the upper lid, and the eye-ball was very prominent, protruding much farther from the orbit than the right. On raising the upper lid, the pupil was found to be fully dilated, and the ball was totally immobile to voluntary efforts, the patient not being able to turn it, even to the slightest extent, in any direction. By pressure upon the ball it would recede somewhat into the orbit. Ophthalmoscopic examination of the fundus showed the media to be clear, the optic disc pale, and the vessels diminished in size. The vision was entirely lost, the brightest light being imperceptible. General sensation of the ball and of the integument of the lids and forehead of the corresponding side did not seem to be impaired.

It is quite difficult for me to understand how the nerves within the orbit could be so injured as to thus completely paralyse every muscle within the orbit, and the circular fibers of the iris and the ciliary muscle, destroy the function of the optic nerve, and not impair the nerves of general sensation, and the sympathetic fibers, which, unopposed, cause the pupil to dilate. It seems, however, that the point of the umbrella-stick may have entered the orbit, and at its apex injured the nerves, whose functions are lost, although the history of the case does not make this absolutely certain.

Upon any theory of the *modus operandi* of the injury and paralysis, the case is interesting and unusual, as presenting the combined symptoms of proptosis, loss of vision, and total paralysis of all the extra- and intra-ocular muscles.

PATHOLOGICAL SPECIMENS

were then exhibited, Dr. M. HARTWIG showing a *calcarious placenta ;* an extirpated *cancerous uterus* from a woman who was, so far, recovering from the operation ; a *benign tumor* removed from the uterus ; and a tape-worm.

Dr. F. W. BARTLETT showed a placenta from a syphilitic woman, and a gall-bladder, filled with concretions, from a patient who, during life, suffered from peculiar gastric and general symptoms.

Dr. LUCIEN HOWE showed an eye which he had removed containing a large piece of cartridge.

